# Anatomic and functional masseter muscle adaptation following orthognathic surgery—MRI analysis in 3 years of follow-up

**DOI:** 10.1186/s40902-024-00437-6

**Published:** 2024-07-19

**Authors:** Fernando Duarte, João Neves Silva, Carina Ramos, Colin Hopper

**Affiliations:** 1 OMD (Portuguese Dental Association), Porto, Portugal; 2 Eastman Dental Institute - University College of London, London, UK; 3 Universitá Cattolica del Sacro Cuore, Rome, Italy; 4 Clitrofa, Centro MédicoDentário e Cirúrgico, Avenida de Paradela 622, Trofa, 4785-248 Portugal; 5 ISAVE - Instituto Superior de Saúde, Amares, Portugal; 6 Interdisciplinary Center for Health Sciences (ICHS), ISAVE - Instituto Superior de Saúde, Amares, Portugal; 7 IUCS – Instituto Universitário de Ciências da Saúde, Gandra, Portugal; 8 Oral & Maxillofacial Surgery Department, UCL Eastman Dental Institute, London, UK

**Keywords:** Orthognathic surgery, Masseter muscle fibres, MRI analysis

## Abstract

**Background:**

Orthodontic and surgical technical advances in recent years have resulted in treatment opportunities for a whole range of craniofacial skeletal disorders either in the adolescent or adult patient. In the growing child, these can include myofunctional orthodontic appliance therapy or distraction osteogenesis procedures, while in the adult, the mainstay approach revolves around orthognathic surgery.

The literature agrees that for a change in craniofacial morphology to remain stable, the muscles acting upon the facial skeleton must be capable of adaptation in their structure and, therefore, their function. Failure of the muscles to adapt to the change in their length or orientation will place undesirable forces on the muscle attachments leading to potential instability of the skeleton. Adaptation can occur through various processes including those within the neuromuscular feedback mechanism, through changes within muscle structure or through altered muscle physiology, and through changes at the muscle/bone interface.

It is now accepted that because there is no single method of assessing masticatory function, several measures should be taken, and whenever possible, simultaneously.

**Methods:**

This investigation was designed to apply several, newly developed and more sophisticated methods of measuring muscle structure and function to a situation where adaptation of muscle is pivotal to the success of a therapeutic approach. Patients attending the combined orthodontic/orthognathic surgery clinic at the Clitrofa – Centro Médico, Dentário e Cirúrgico, in Trofa, Portugal, were screened. Ten patients scheduled for a bimaxillary osteotomy involving a combination of maxillary Le Fort I impaction procedure coupled with a sagittal split advancement of the mandible were selected to form the study group.

The patients have MRI of the masseter muscle to evaluate the masseter muscle volume and fibre orientation changes. This exam was taken before surgery (T0), 6 to 12 months after surgery (T1), and 3 years after surgery (T2), by two independent observers, according to the protocol jointly developed between the Eastman Dental Institute – University of London and the MRI Centre - Department of Radiology at John Radcliffe Hospital – University of Oxford.

**Results:**

Significant differences (*p* < 0.05) have been identified between Time 0 (pre-op) and Time 1 (6–12 months post-op) regarding the masseter area (mm^2^). The differences against Time 0 (pre-op) seem to disappear at Time 2 (3 years post-op).

**Conclusions:**

MRI therefore seems to be a valid tool for measuring differences in the masseter muscle area and volume associated with high-severity occlusal deformities, although showing not to be as efficient in detecting the same differences in cases of low-severity occlusal deformities.

## Background

Changes in masticatory musculature structure and function may be either developmental, as seen in anomalies of vertical facial form, or adaptative, as seen during procedures such as orthognathic surgery and functional appliance orthodontic therapy [1, 2].

The principal goals of orthognathic surgery are the improvement of occlusal relationships, facial esthetics, and function of the masticatory system in patients with dentoskeletal deformities [3, 4].

The results of some studies indicate that patients scheduled for orthognathic surgery will tend to have lower mastication forces than controls [5, 6]. The lower forces in patients, however, do not seem to be the result of lower efficiency in the jaw muscles. Instead, the results of this study suggest that patients, prior to treatment, may experience differences in sensory feedback or have lower motivation to generate large forces [7].

Advances in medical imaging have created ever increasing volumes of complex data obtained from the patient. The interpretation of such information has become a specialty in itself and the surgeon at times may be left bewildered as to how best to apply the available information to the practicalities of physical intervention. The surgeon seeks to understand the exact morphology of the abnormality, its relationships to surrounding anatomy, and the best way to access and correct the pathology operatively. Such specific information is not readily available in the radiologist’s report and however experienced the surgeon may be at interpreting images such questions often cannot be easily answered [8].

Three-dimensional (3D) imaging has been developed to narrow the communication gap between radiologist and surgeon. By using 3D imaging, a vast number of complex slice images can be quickly appreciated. The term “three-dimensional”, however, is not a truly accurate description of these images as they are still displayed on a radiological film or flat screen in only two dimensions. The advent of 3D imaging has not only improved data display but also promoted the development of even more useful technologies to assist the surgeon in diagnosis and planning [8].

## Masseter muscle architecture

The average length of masseter muscle three-layer fibres is 19–30 mm; those in the posterior region are about 35% shorter than those in more anterior. This difference in length is almost certainly related to the need for differential fibre shortening during function, but it is not accounted for entirely by the relative sizes of the respective fibre lever arms measured from the jaw’s function “center” of rotation, and it suggests that fibre tensions may be greater anteriorly than posteriorly as different opening movements are made during function. The masseter also contains at least five intramuscular aponeuroses, some of which descend from the zygomatic arch and interweave with others ascending from the ramus. Fibres pass obliquely between them. These flat tendon sheets can be visualized by magnetic resonance imaging (MRI) in living subjects, and their orientation varies. The motor unit territories are very small in the masseter, and the fibres from each unit tend to remain in close proximity. Differential activation of muscle fibres occurs in various regions of the masseter, causing various fibre collections in the masseter’s mediolateral layers, or between tendon sheets, to contract differentially according to the task. There is probably considerable mechanical diversity within a given muscle and, since each muscle’s structural elements vary from person to person, also equal mechanical diversity between individuals [9].

It is difficult to predict what actually happens internally when the masseter contracts. Depending upon a subject’s morphological type, the task being attempted, and the highly individual contraction strategy used, various groups of muscle fibres will contract and shorten isovolumetrically. As they do so, they thicken, and their transverse diameters will increase. There will be regional changes in muscle thickness, presumably shaped by the relative balance between mutually-contracting fibre groups, thick, layered tendons near the zygomatic arch, and the extent to which tendon sheets move within the muscle. Localized distortion of tendon sheets is possible, and it is likely that regional tensions will be produced at muscle-tendon interfaces, while the net effect may be qualitatively similar between two individuals [10].

To complicate matters, the effects of these changing physical events are themselves uncertain. Different degrees of local intramuscular compression probably alter regional blood flow within the muscle, but presently, there is no evidence describing specifically how vascular physiology in the masseter or any other human jaw muscle is affected selectively by local changes in its physical environment. Apart from any effect on the vascular bed, the production of differential, excessive internal muscle tension, if it follows the same pattern as it does elsewhere in the musculoskeletal system, can lead to local tissue injury. If so, it most probably will occur within the muscle fibres at a short distance from the muscle-tendon interface rather than at the interface itself. Finally, any excessive loading of tendons per se can result in persistent, local inflammation as is commonly found in other skeletal muscles. Any of these hypothetical events would cause biochemical changes in the masseter. The changes would be local and include the release of algesic chemicals [11].

## Magnetic resonance imaging

MRI is a non-invasive imaging technique that is one of the most promising and leading imaging modalities for the diagnosis of diseases and other conditions in the head and neck region [12]. A major advantage of MRI over conventional X-ray imaging is the high soft tissue contrast, which allows much better visualization of specific anatomical structures (e.g. nerves, blood vessels) using magnetic fields without exposing patients to ionizing radiation [12]. Despite the limitations in hard tissue imaging, MRI has advanced rapidly over the past two decades with various technical innovations and advanced imaging protocols, offering a wide range of new diagnostic capabilities in dentistry [12, 13].

MRI scans provide the best definition of facial muscles when segmented from DICOMs [13, 14]. Previous works on facial tissue characterization have demonstrated that different areas of facial soft tissues have different biomechanical properties in terms of longitudinal tissue stiffness (Young’s modulus–E) and transverse behavior (Poisson’s ratio–ν) [13, 14].

The residual limitations of MRI in the oral cavity are susceptibility to motion artifacts, complex anatomic courses of small-sized blood vessels and nerves, and image distortion and artifacts due to magnetic field inhomogeneities caused by metallic dental restorations [13].

A customised software programme has been developed at John Radcliffe Hospital - Oxford University which enables the reconstruction of 3D images allowing measurement of muscle volume and area with a high level of accuracy.

To date, this technology had only been applied to tongue muscles, and when applied to the muscles of mastication, the resolution and results were disappointing.

The goal was to develop the system and software to produce accurate and reproducible data for masticatory muscles which not only provided data for muscle area and volume, but also was of sufficient detail to enable analysis of muscle fibre orientation in particular of masseter muscle.

The masseter muscle displays a penniform structure typically characterized by the presence of alternating muscular/aponeurotic layers. The anatomical sections and the MRI section in the same plane allowed the appearance of the intra-muscular aponeurotic layers on the MRI to be defined [15].

## Methods

### Research design

The present study is an observational prospective study with quantitative methodology.

### Sample

A study group of 10 patients attending the combined orthodontic/orthognathic surgery clinic at the Clitrofa – Centro Médico, Dentário e Cirúrgico, in Trofa, Portugal, were selected for the present study by a convenience non-probability sampling method. All the selected patients present skeletal class III malocclusion characterized by a concave facial profile with lower lip protrusion or upper lip retrusion or a combination of the two. The most consistent characteristics of skeletal class III malocclusion seem to be the dental Angle’s class III canines and molars, the presence of anterior cross-bite, and retroclined mandibular incisors.

During the sequential MRI image period, all patients received ear protectors and were instructed to maintain a relaxed muscle posture and closed jaw position (maximal intercuspal position of the lower jaw).

The patients scheduled for a bimaxillary osteotomy involving a combination of maxillary Le Fort I impaction procedure coupled with a sagittal split advancement of the mandible were selected to form the study group. Vertical moves of 2 mm for minor, 4 mm for intermediate, and 6 mm for major impactions are appropriate for all cases. These three categories also simplify the decision-making process. Before surgery, all patients signed their informed consent form.

The inclusion criteria are as follows: All patients presenting at joint orthodontic/orthognathic clinic purposed to orthognathic surgery and that accept the treatment. Diabetic patients were included but noted.

The exclusion criteria are as follows: Patients who gave a history of myopathies, endocrine disorders, connective tissue disorders, autoimmune diseases, bone disease, bleeding disorders, and regular use of prescribed drugs were excluded from the study.

Osteotomies were performed using piezoelectric surgery that is based on the use of ultrasound. It offers precise bone cuts without damaging any soft tissue, minimizing the invasiveness of surgical procedure, and the opportunity of working in a field which is almost totally blood-free. It reduces the impact on soft tissues (vessels and nerves) which lie adjacent to the areas of treatment.

Maxillomandibular fixation (MMF) was performed with surgical archwire fixation, L-shaped osteosynthesis plates, and self-tapping screws. Postoperative orthodontic treatment lasted an average of 6 months. The final occlusion should provide unhindered closure in centric relation, smooth-sliding lateral and protrusive movements, and an optimal bilateral vertical contact dimension.

### Data collection instruments

The anatomical and functional heterogeneities of the masseter muscle may influence the spatial differences in muscle thickness. For the sake of systematization and reduction of variables, it was decided to use only the left masseter muscle.

MRI technique was used to measure masseter muscle volume and fibre orientation changes in the selected patients. This evaluation was taken before surgery (T0), 6 to 12 months after surgery (T1), and 3 years after surgery (T2), by two independent observers, according to the protocol jointly developed between the Eastman Dental Institute – University of London and the MRI Centre - Department of Radiology at John Radcliffe Hospital – University of Oxford. It should be considered that during the different evaluation periods, the patients’ occlusion changed, namely, T0-skeletal class III, T1-skeletal class I, and T2-skeletal class I.

### Anatomics™ software

The Anatomics™ Rx software is a 3D DICOM viewer and allows to view CT and MRI scan data in both slice format and fully interactive 3D. Anatomics™ can convert 3D images to the STL format for rapid prototyping, or as a bridge from medical imaging to computer-aided design (CAD). A good quality 3D scan is required to create an accurate biomodel or implant.

To standardize the scanning process, a scanning protocol was developed and applied that describes the preferred imaging parameters and provides the imaging technician with an area to note specifics. The patient must remain completely still during the scan; if the patient moves during the scan, it will need to be repeated. Only the original fine-slice data must be used in the software, reformats will not be accepted. Fine overlapping slices must be used, the thickness of 1 mm (or nearest to) and a spacing of 0.8 mm.

The objective was to extract the muscle from the image (margins identification, extract the muscle considering the 3 planes of space, calculation of area and volume). The software allows the correction of limits at any time which gives the observer the capacity to double-check all the processes.

During this study, the MRI machine used was a Sigma MR/I Twinspeed from GE Medical Systems; after several attempts, the software was further developed to produce slices through the muscle at 1-mm intervals rather than 2 mm; the scanning time was about 7 min.

**Fig. 1:**
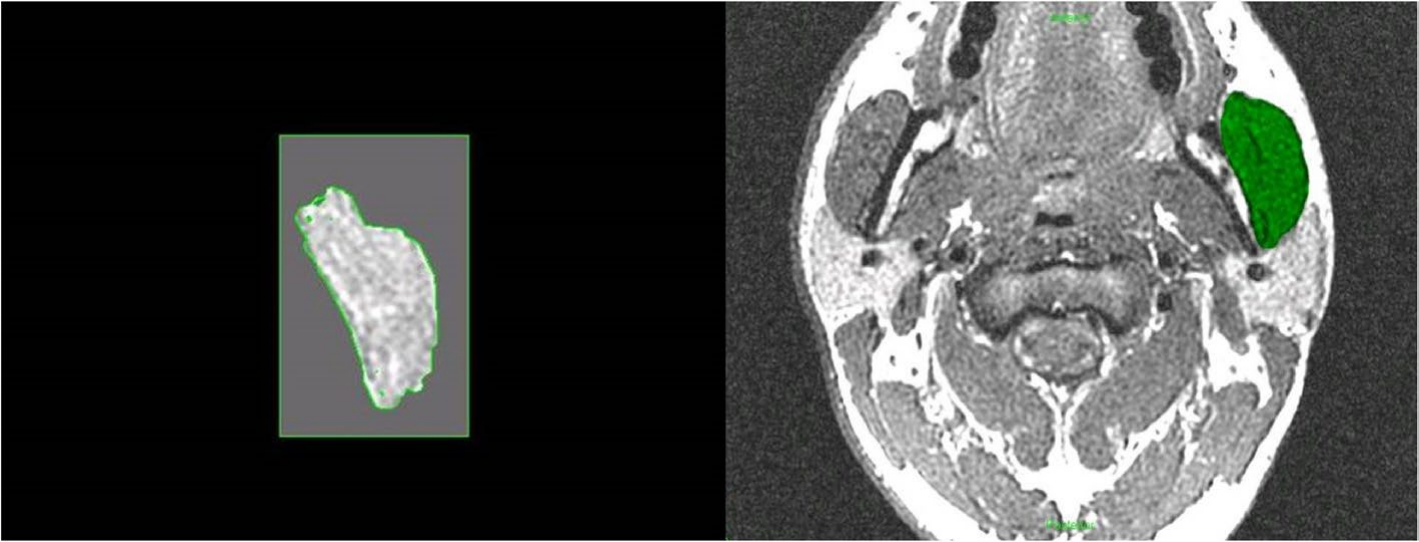
Identification of masseter muscle limits in a sagittal plane

**Fig. 2:**
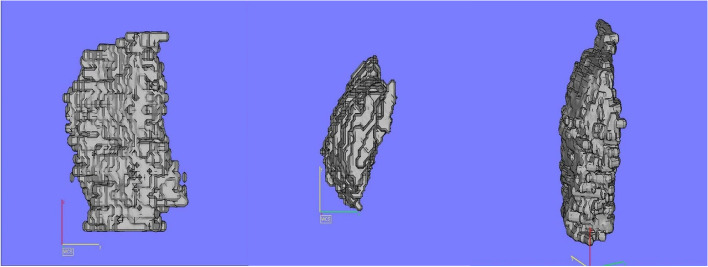
Final images from the left masseter muscle reconstruction using Anatomics™ software

The first masseter muscle 3D image reconstruction was acceptable in terms of definition, area, and volume but with a lack of detail in terms of muscle fibre visualization and orientation (Fig. 1). Increasing the scanning time from 5 to 7 min and changing the muscle slices to 1-mm intervals was possible for the acquisition of more muscle details. As a consequence, the resolution of the muscles was greatly enhanced, and the final masseter muscle 3D image reconstruction permits a good visualization of muscle fibres and their orientation (Fig. 2). This type of reconstruction has also allowed visualization of the muscle’s bony attachments and enabled the measurement of potential changes in orientation in relation to a static landmark unaffected by surgery (e.g. Frankfort plane) or in relation to functional identifiers (e.g. occlusal plane).

### Experimental procedure

**Fig. 3:**
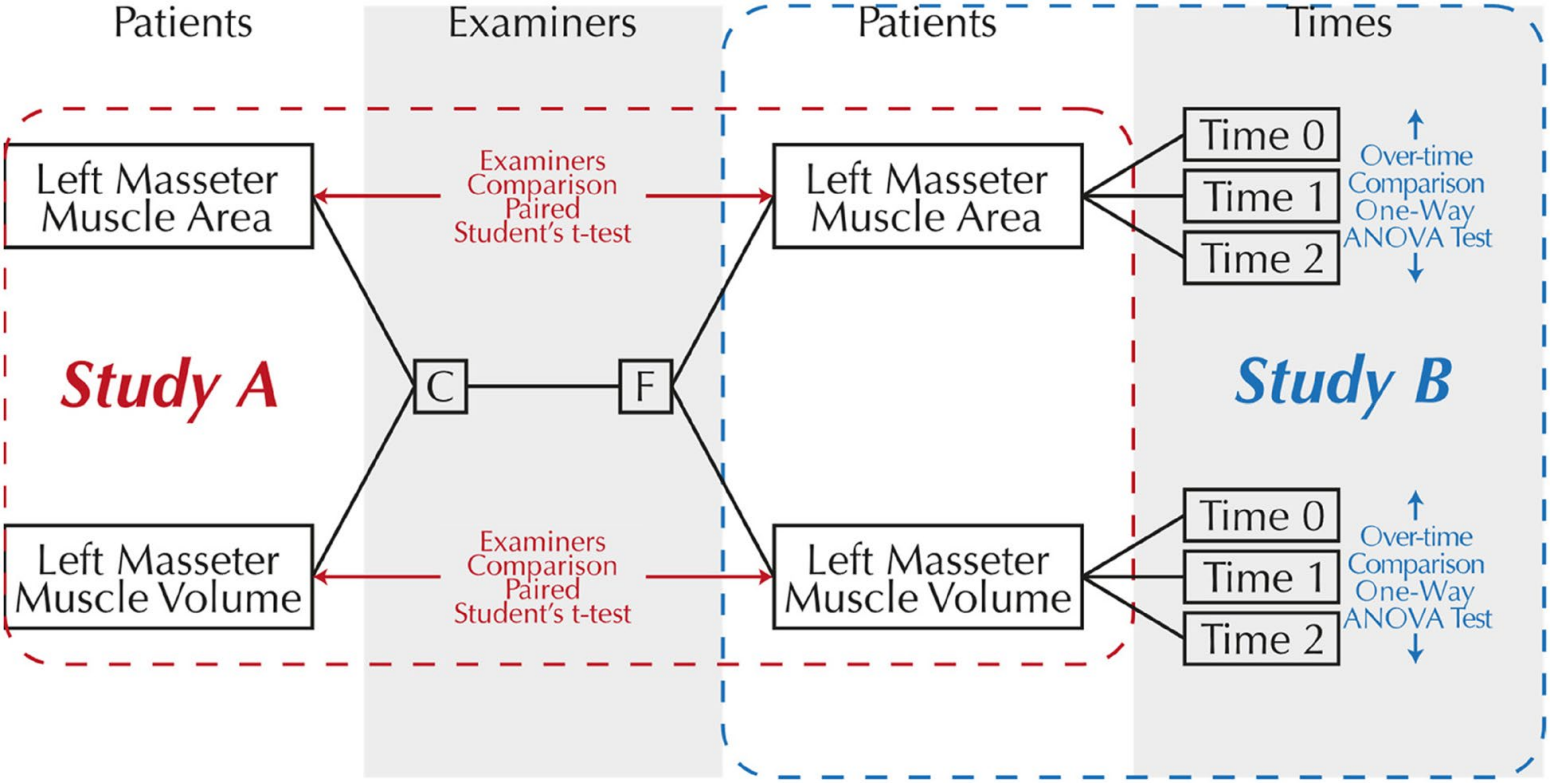
Experimental design used in the present work

The experimental design used for this work is depicted in Fig. 3 and involves two different studies: Study A, which investigates the effect of examiner change on the measurement of left masseter muscle area (mm^2^) and left masseter muscle volume (mm^3^) of ten different patients by two independent observers; and Study B, which investigates the variation of left masseter muscle area (mm^2^) and left masseter muscle volume (mm^3^) in three different times: before surgery (T0), 6 to 12 months after surgery (T1), and 3 years after surgery (T2).

#### Study A—Effect of examiner (F or C) change on the measurement of left masseter muscle area (mm^2^) and left masseter muscle volume (mm^3^) of ten selected patients

Research question: Are there any significant statistical differences between examiners F and C regarding the measurement of mean left masseter area (mm^2^) and left masseter volume (mm^3^) of ten patients by MRI?

- H0: There are no significant statistical differences between examiners F and C regarding the measurement of mean left masseter area (mm^2^) and left masseter volume (mm^3^) of ten patients by MRI.
- H1: There are significant statistical differences between examiners F and C regarding the measurement of mean left masseter area (mm^2^) and left masseter volume (mm^3^) of ten patients by MRI.

#### Study B—Effect of time (Time 0, pre-op versus Time 1, 6–12 months post-op versus Time 2, 3 years post-op) on the left masseter muscle area (mm^2^) and left masseter muscle volume (mm^3^) of ten selected patients

Research question(s): Are there any differences between the left masseter muscle area (mm^2^) and left masseter muscle volume (mm^3^) of the ten selected patients over time (Time 0, pre-op; Time 1, 6–12 months post-op; and Time 2, 3 years post-op).

- H0: There are no differences between the left masseter muscle area (mm^2^) and left masseter muscle volume (mm^3^) of the ten selected patients over time (Time 0, pre-op; Time 1, 6–12 months post-op; and Time 2, 3 years post-op).
- H1: There are differences between the left masseter muscle area (mm^2^) and left masseter muscle volume (mm^3^) of the ten selected patients over time (Time 0, pre-op; Time 1, 6–12 months post-op; and Time 2, 3 years post-op).

## Statistical analysis

IBM® SPSS® version 25 was used to analyze the data obtained in the present work. The data were first tested to ensure they conformed to a normal distribution by using Kolmogorov-Smirnov test. The data were then tested to ensure they complied with variance homogeneity by using the Levene test.

Descriptive statistics measures included the arithmetic mean (x ®) and standard deviation (SD) if the data were normally distributed and the variance was constant. Where the data were not normally distributed nor the variance was constant, the median and the inter-quartile range (IQR) were noted.

Where the requirements for parametric statistical analysis were met, inferential analysis of examiner comparison in Study A involved the use of paired two-tailed Student’s *t*-test. In the same conditions, the inferential analysis of times comparison in Study B involved the use of one-way analysis of variance (ANOVA).

Where the requirements for parametric statistical analysis were not met, inferential analysis of examiner comparison in Study A involved the use of the Wilcoxon signed rank (U) test for paired data. In the same conditions, inferential analysis of times comparison in Study B involved the use of the Kruskal-Wallis (H) test.

Where statistically significant differences were found by one-way ANOVA test, the multiple-comparison post hoc Bonferroni test was performed to identify the pairs of categories where the statistically significant differences were located.

The minimum level of significance (α level) accepted throughout the development studies was 0.05 (*), considered to be moderately significant. Levels of 0.01 (**) were considered significant and 0.001 (***) was designated as highly significant. A lack of statistical significance was designated as ns.

## Results

In order to make the presentation of results easier to understand, they were subdivided into two items: effect of examiner selection and effect of time. Because one-way ANOVA only gives information about the presence of differences, not specifying where these differences are located, a post hoc Bonferroni test was used to perform pairwise comparison regarding the time points.

### Study A—Effect of examiner (F or C) change on the measurement of left masseter muscle area (mm^2^) and left masseter muscle volume (mm^3^) of ten selected patients

The following Table 1 presents the mean left masseter areas (mm^2^) and mean left masseter volumes (mm^3^) of ten selected patients measured by two independent examiners (F and C).

**Table 1:** Mean left masseter area (mm^2^) and mean left masseter volume (mm^3^) of ten patients measured by independent examiners F and C. Data was obtained by MRI

**MRI analysis parameter**	**Examiner F**	**Examiner C**
**Left masseter area (mm**^**2**^**) (average ± SD)**	12,034.77 ± 998.32	12,039.40 ± 997.30
**Left masseter volume (mm**^**3**^**) (average ± SD)**	29,939.83 ± 3104.06	29,954.67 ± 569.333

The statistical comparison between examiners F and C regarding the measurement of mean left masseter area (mm^2^) and mean left masseter volume (mm^3^) of ten patients by MRI was performed using a paired Student’s *t*-test, and the results are presented in the following Table 2.

**Table 2:** Statistical parameters obtained in the paired Student’s *t*-test for the comparison of examiners F and C regarding the measurement of mean left masseter area (mm^2^) and mean left masseter volume (mm^3^) of ten patients by MRI

**Examiner comparison**	Mean difference*	**Standard deviation of differences**	**Degrees of freedom *(df)***	**Test statistic from paired *t*-test**	***P*-value from paired *t*-test**
**Examiner F versus examiner C, left masseter muscle area (mm**^**2**^**)**	−4.633	79.466	29	−0.319	0.752
**Examiner F versus examiner C, left masseter muscle volume (mm**^**3**^**)**	−14.833	94.659	29	−0.858	0.398

^*^The mean difference is significant at the 0.05 level

### Study B—Effect of time (Time 0, pre-op versus Time 1, 6–12 months post-op versus Time 2, 3 years post-op) on the left masseter muscle area (mm^2^) and left masseter muscle volume (mm^3^) of ten selected patients

The following Table 3 presents the variation of mean left masseter area (mm^2^) and mean left masseter volume (mm^3^) of ten selected patients over time (Time 0, pre-op; Time 1, 6–12 months post-op; and Time 2, 3 years post-op).

**Table 3:** Variation of mean left masseter area (mm^2^) and mean left masseter volume (mm^3^) of ten patients measured over time (Time 0, pre-op; Time 1, 6–12 months post-op; and Time 2, 3 years post-op)

**MRI analysis parameter**	**Time 0 (pre-op)**	**Time 1 (6–12 months post-op)**	**Time 2 (3 years post-op)**
**Left masseter area (mm**^**2**^**) (average ± SD)**	12,511.60 ± 864.22	11,648.40 ± 986.73	11,951.25 ± 956.54
**Left masseter volume (mm**^**3**^**) (average ± SD)**	31,114.85 ± 2851.57	29,116.70 ± 3234.68	29,610.20 ± 2945.05

The statistical comparison between the three time points (Time 0, pre-op; Time 1, 6–12 months post-op; and Time 2, 3 years post-op) regarding the left masseter muscle area (mm^2^) and left masseter muscle volume (mm^3^) by MRI of the ten selected patients was performed using a one-way ANOVA test, and the results are presented in the following Table 4.

**Table 4:** Statistical parameters obtained in the one-way ANOVA test for the statistical comparison between the three time points (Time 0, pre-op; Time 1, 6–12 months post-op; and Time 2, 3 years post-op) regarding the left masseter muscle area (mm^2^) and left masseter muscle volume (mm^3^) by MRI

**Examiner comparison**	**Degrees of freedom *(df)***	**Test statistic from paired one-way ANOVA test**	***P*-value from paired *t*-test**
**Time 0, pre-op versus Time 1, 6–12 months post-op versus Time 2, 3 years post-op, masseter muscle area (mm**^**2**^**)**	2 / 57	4.367	0.017*
**Time 0, pre-op versus Time 1, 6–12 months post-op versus Time 2, 3 years post-op, masseter muscle volume (mm**^**3**^**)**	2 / 57	2.364	0.101

^*^The mean difference is significant at the 0.05 level

### Post hoc Bonferroni test

**Study A:** The results show no significant statistical differences between examiner F and examiner C regarding the measurement of the left masseter area (mm^2^) and left masseter volume (mm^3^) of the ten selected patients through MRI, when the measurement is made in the same experimental conditions (*p* > 0.05).

In view of these results, the change of examiner is not a factor that influences the measurement of left masseter area (mm^2^) and left masseter volume (mm^3^).

**Study B:** The results show significant differences in the left masseter muscle area (mm^2^) over time (*p*-value = 0.017), although these differences have not been identified regarding the left masseter muscle volume (mm^3^) (*p*-value > 0.05).

Because one-way ANOVA only gives information about the presence of differences, not specifying where these differences are located, a post hoc Bonferroni test was used to perform pairwise comparison regarding the time points regarding the left masseter muscle area (mm^2^) of the ten selected patients, and these results are represented in the following Table 5.

**Table 5:** Statistical parameters obtained in the post hoc Bonferroni test for the comparison of the different time points (Time 0, pre-op; Time 1, 6–12 months post-op; and Time 2, 3 years post-op) regarding the mean left masseter area (mm^2^) of ten selected patients

**Independent variable**	**Mean difference (I-J)**	**Std. error**	**Sig.**
**Time 0 (pre-op)**			
**Time 1 (6–12 months post-op)**	863.200	296.394	0.015^*^
**Time 2 (3 years post-op)**	560.350	296.394	0.191
**Time 1 (6–12 months post-op)**			
**Time 0 (pre-op)**	−863.200	296.394	0.015^*^
**Time 2 (3 years post-op)**	−302.850	296.394	0.934
**Time 2 (3 years post-op)**			
**Time 0 (pre-op)**	−560.350	296.394	0.191
**Time 1 (6–12 months post-op)**	302.850	296.394	0.934

*The mean difference is significant at the 0.05 level

## Discussion

Altered muscle function is implicated in the aetiology of vertical facial deformities. The contractile properties of muscle are largely determined by a number of different isoforms of myosin heavy chain (MyHC), and the pattern of MyHC gene expression is one measure of the phenotype and functional potential of a muscle [16].

Two extremes of vertical facial form have been described, long face syndrome and short face syndrome [17]. The long face syndrome (LFS) is characterized by the clinical and radiographic features of increased lower anterior face height, increased maxillary/mandibular plane angle, increased gonial angle, and tendency to anterior open bite. The short face syndrome (SFS) exhibits the reverse of these features. The differences between the two syndromes reflect their divergent growth patterns, where LFS subjects exhibit a downward and posterior growth rotation of the mandible, and SFS subjects exhibit an anterior growth rotation [18]. A significant proportion of the patients presenting with extreme vertical facial discrepancies require surgery to correct their jaw relationship [19, 20].

It has been proposed that the muscles of mastication are important determinants of vertical facial growth [21]. Studies of masseter muscle function have shown significant differences between LFS and SFS subjects with respect to electromyographic (EMG) activity and the magnitude of maximum voluntary bite force; SFS subjects demonstrate higher EMG activity and exert greater bite forces than LFS subjects [22], whether the observed differences in muscle function are primary causal factors or are secondary to the development of vertical facial form [23]. Furthermore, changes in vertical facial form have been induced by either increasing or decreasing the normal activity of the elevator muscles during postnatal growth [21, 24].

The molecular motors of muscle are the myosin heavy chains (MyHC) located in the myofibrillar apparatus of muscle fibres [25]. Muscle fibres are the functional, contractile components of muscle, and the physiological properties of these fibres are largely determined by a number of different MyHC isoforms variously distributed between fibres with different contractile properties [25].

The masseter differs from somatic skeletal muscle in the range of MyHC isoforms expressed in the adult muscle [26]. The myosin heavy chains are encoded by a multigene family, and the major adult isoforms expressed in human skeletal muscle are the slow or ß-cardiac, IIa, and IIx MyHCs that are expressed in the type I, type IIa, and type IIb fibres, respectively [25]. A human homologue to the IIb MyHC isoform described in the rat and other species has yet to be identified [27]. Additionally, the adult human masseter expresses embryonic, perinatal, and α-cardiac MyHCs [28].

The few studies of the distribution of fibre type in the muscles of subjects with extremes of vertical facial form suggest that the contribution of different fibre components to the masseter phenotype overall may vary between normal subjects and those with vertical facial deformity (VFD). Comparisons of the fibre-type distribution and cross-sectional areas in biopsies of the anterior deep masseter have revealed a reduced contribution of type II fibres to the total percentage cross-sectional area in LFS subjects [29]. However, the masseters of SFS subjects have demonstrated either no differences from a control group or an increased type II fibre contribution in the same region of the muscle [29].

The differential increase in anterior and posterior face heights produced at surgery may not only stretch the muscle attachments but also change the orientation of the muscle fibres to the occlusal plane. Adaptation would be necessary with regard to the resting length and also in relation to altered functional activity. It has been noted that such adaptation may occur up to 12 months following surgery [30]. In a study of Hunt and Cunningham [30], surgical alteration of the vertical facial heights was accompanied by an immediate adaptation of the clinical freeway space, presumably mediated through the proprioceptive system. The physiological rest position can be identified by eliminating the sensorimotor feedback from the teeth, so allowing the mandible to adopt a posture dependent upon the resting length of the elevator muscles is partially adapted to the skeletal change immediately following operation, but continued to adapt up to 12 months post-surgery, especially in the vertical excess patients [30].

Any increase in posterior vertical facial dimension is prone to relapse in the long term. At least three possibilities exist as to how this may occur. Firstly, stretching of the pterygo-masseteric sling could lead to increased pressure at the osteotomy site with subsequent bone resorption and loss of vertical dimension. Secondly, in an attempt to maintain an efficient muscular system, both at rest and during function, muscle adaptation could occur through migration of the attachments in preference to increasing the number of sarcomeres. As a consequence, the area of bone devoid of attachment could remodel or resorb thereby reducing the vertical height. Thirdly, a combination of these two hypotheses could exist [30].

The architecture of the masseter muscle has been studied for a long time, but the lack of clinical applications led to descriptions which were often global or contradictory, giving the muscle sometimes two bundles sometimes three. The successive studies of Gaspard [31–33], Yoshikawa [34, 35], and Gaudy [36] allowed the definition of the arrangement of the muscular aponeurotic layers making up the human masseter muscle. Unger [37] affirmed the value of magnetic resonance imaging in the oro-facial field for the study of the musculature of the tongue and the walls of the oral cavity, but gave only very general information on the masticatory muscles [38].

Several studies investigated the changes in the size and masticatory force of the masticatory muscles after orthognathic surgery. Katsumata et al. indicated that in mandibular prognathism, the cross-sectional area of the masses decreases after 3 months of mandibular setback but shows a tendency to return to normal after 1 year [39]. In addition, Ueki et al. reported that there are no significant differences in the cross-sectional area of the masseter in mandibular prognathism 1 year after SSRO in comparison with the preoperative area [40]. Trawitzki et al. also reported that when mandibular setback was conducted on patients with a class III dentofacial deformity, the thickness of the masseter muscle increased [41]. The study of Kanga et al. showed that the volume-to-length ratio of the masseter and lateral pterygoid muscles at 1 year after the mandibular setback did not show a significant difference compared with the preoperative value [42].

In a study with 30 skeletal class III patients with dentofacial deformities, 17 were treated by sagittal split ramus osteotomy with rigid osteosynthesis, and 13 were treated by intraoral vertical ramus osteotomy without osteosynthesis; Katsumata et al. reported that masseter muscle crosssectional area was lower in the group who underwent sagittal split ramus osteotomy and intraoral vertical ramus osteotomy. The evaluation was done using three-dimensional CT imaging [39].

Kikuta et al. reported that occlusal force was decreased 3 months after orthognathic surgery, but increased 6 months after the surgery [43]. The results of this study suggest that particular attention should be paid to masseter muscle atrophy in patients with worse open bite after preoperative orthodontic treatment and in those with maxillary undergrowth. However, it is not clear if masticatory ability would be compromised by masseter muscle atrophy immediately after the surgery.

Decreased maximum occlusal force in patients with open bite has been reported, which supports results that increased open bite led to decreased masseter muscle cross-sectional area [44, 45].

## Conclusions

A number of studies have reported increased bite force, occlusal contact area, and EMG activity and improved masticatory efficiency after surgery; however, the reason for this improvement is unclear [7]. Previous studies reported that the postoperative improvements in muscular activity were due to better occlusal stability and not to surgically induced biomechanical advantages [46, 47]. The importance of occlusion for the neuromuscular equilibrium and dental supports was investigated in patients undergoing orthognathic surgery. Changes in muscle size; increased occlusal contact area providing greater dental support; sensitivity of teeth, muscles, and the temporomandibular joints; and even the patients’ willingness to exert maximum effort have been suggested as factors in determining the occlusal force after surgery [7].

The continuous changes in masseter muscle size in our study indicate that not only was the skeletal environment altered by surgery, but additional adaptation to new stomatognathic environments also occurred over time with improved occlusion and masticatory activity by orthodontic treatments.

Significant differences (*p* < 0.05) have been identified between Time 0 (pre-op) and Time 1 (6–12 months post-op) regarding the mean left masseter area (mm^2^).

It is interesting to notice, however, that the differences against Time 0 (pre-op) seem to disappear at Time 2 (3 years post-op), which may reveal the long-term decrease in the area of mean left masseter area (mm^2^) or relapse.

An adequate sample makes the investigation more efficient: the data generated is reliable, and the investment of resources is as limited as possible, while at the same time complying with ethical principles. The use of the sampling design directly influences the research results. The sample of 10 patients reveals that this is an uncommon type of surgery, carried out in the vast majority of cases in private health services and requiring the patient’s economic power.

MRI therefore seems to be a valid tool for measuring differences in the masseter muscle area and volume associated with high-severity occlusal deformities (maxillary Le Fort I impaction of 6 mm), although showing not to be as efficient in detecting the same differences in cases of low-severity occlusal deformities (maxillary Le Fort I impaction of 2 mm for minor and 4 mm for intermediate cases).

Future studies comprising larger samples of patients and other different methods of measuring changes in masticatory muscle structure and function are currently being equated to measure the efficacy of orthognathic surgery.

## Data Availability

Please contact the author for data requests.

## Abbreviations

3D: Three-dimensional
MRI: Magnetic resonance imaging
MyHC: Myosin heavy chain
VFD: Vertical facial deformity
CAD: Computer-aided design
SD: Standard deviation
IQR: Inter-quartile range
